# Atosiban improves the outcome of embryo transfer. A systematic review and meta-analysis of randomized and non-randomized trials

**DOI:** 10.5935/1518-0557.20200016

**Published:** 2020

**Authors:** Juan Enrique Schwarze, Javier Crosby, Antonio Mackenna

**Affiliations:** 1 Reproductive Medicine at Clinica Las Condes, Santiago, Chile; 2 Obstetrics and Gynecology Department, Universidad de Santiago, Chile

**Keywords:** atosiban, *in vitro*, pregnancy rate

## Abstract

**Objective:**

To estimate the effectiveness of Atosiban in improving the outcome after embryo transfer. The effectiveness of embryo transfer per cycle is still relatively low. One possible explanation might be uterine contractility that expels the transferred embryos. Atosiban improved the outcome of embryo transfer by reducing uterine contractility.

**Methods:**

Data sources: A systematic review of papers in English using MEDLINE and EMBASE (1990-2019). Search terms included Atosiban, embryo transfer. Study selection: We included studies that compared the outcomes of embryo transfer with Atosiban and a control group. Data Extracting: Independent extraction of papers by two authors, using predefined data fields, including study quality indicators.

**Results:**

All pooled analyses were based on a fixed-effect model. Four randomised controlled trials, including 1,025 women, and two non-randomised trials, including 686 patients, met our inclusion criteria. In both studies, the heterogeneity was moderate. Atosiban increased clinical pregnancy rates regardless of the indication for ART or type of embryo transferred. Pooled OR in randomized controlled trials reached 1.47 (1.18-1.82), and in non-randomised controlled trials it reached 1.50 (95% CI 1.10-2.05)

**Conclusion:**

Atosiban appears to increase the clinical pregnancy rates in women undergoing embryo transfer.

## INTRODUCTION

In spite of advanced progress in assisted reproduction technology (ART) over the past 20 years, the effectiveness of embryo transfer (ET) per cycle is still relatively low. In 2015, the delivery rate (DR) per ET in Latin America reached 25.6% in fresh autologous ET, and 36.8% when using donated eggs (Zegers-Hochschild *et al*., 2017a;b).

After ET the effectiveness of embryo implantation depends on embryo quality, endometrial receptivity and adequate dialogue between them (Achache & Revel, 2006). Traditionally, an abnormal chromosomal complement has been considered as the main cause for implantation failure and, in clinical practice, considerably little effort has been devoted to improve uterine receptivity. Generally, appropriate endometrial status, sufficient endometrial perfusion and absence of excessive uterine contractions are necessary for ideal endometrial receptivity and to facilitate embryo implantation (Pierzynski & Reinheimer, 2007). Although increased contractions have been found in approximately 30% of patients undergoing ET, to date uterine contractility is not included in any diagnostic measures, and the therapies to reduce uterine contractions before ET such as beta agonists, non-steroid anti-inflammatory drugs (NSAIDs) or progesterone had not shown definite benefits (Bernabeu *et al*., 2006; Fanchin *et al*., 2001).

Theoretically, uterine contractions can expel the embryos after transfer, as per indicated by a study of mock embryo transfer processes (Fanchin *et al*., 1998a). As such, a stepwise decrease in implantation rates and clinical ongoing pregnancy rates occurred from the lowest to the highest uterine contraction frequencies (Fanchin *et al*., 1998b).

Atosiban was administered to inhibit uterine contractions (He *et al*., 2016a; Hebisha *et al*., 2016). Atosiban is a uterine-specific, mixed vasopressin V1-a and oxytocin-receptor antagonist, that is registered for tocolysis in imminent premature birth. It also inhibits uterine contractility in nonpregnant women. Thus, Atosiban may decrease uterine contractions and promote uterine receptivity in patients undergoing embryo transfer.

We conducted this systematic review and meta-analysis to investigate whether Atosiban improves pregnancy outcomes in the women undergoing ET.

## MATERIALS AND METHODS

### Literature search and study selection

We searched the computerised databases Medline and Embase from January 1990 to July 2019. We explored the following terms as free text terms and MeSH terms (shown in italics): *(embryo transfer; atosiban)* and (*fertilization in vitro; atosiban*). Additionally, the citation lists of all relevant publications and review papers were hand-searched.

### Selection criteria, data extraction and quality assessment

We established the criteria for inclusion/exclusion of studies prior to the literature search. We selected randomised controlled trials and observational studies that compared Atosiban at the time of ET with placebo or no treatment. Trials that included intracytoplasmic injection of sperm as well as *in vitro* fertilization were eligible, as were studies using fresh and frozen/thawed ET. We excluded trials that evaluated other intervention in conjunction with Atosiban. We imposed no restrictions on publication type (that is, either full article or abstract), and restricted language to English. Two authors (JES and JC) independently selected articles and extracted data, with disagreements resolved by discussion.

### Outcome measures

The pre-specified primary outcomes were clinical pregnancy (that is, presence of at least one gestational sac or foetal heartbeat, confirmed by transvaginal ultrasound) and live birth.

### Risk of publication bias

For each trial, we plotted the effect by the inverse of its standard error. The symmetry of such ‘funnel plots’ was assessed visually and formally analyzed to help understand whether the results of their review are robust, all of which should be reported. Such analyses include sensitivity analysis, subgroup analysis, and meta-regression.

### Risk of bias assessment

We evaluated the methodological quality of trials using the Cochrane risk of bias tool (Sterne *et al*., 2016; Higgins *et al*., 2011). The items evaluated in randomized trials were: concealment of randomisation sequence allocation (selection bias), allocation concealment (selection bias), blinding of participants and personnel (detection bias), incomplete outcome data (attrition bias), selective reporting (reporting bias) and other biases (Higgins *et al*., 2011). In the case of non-randomized trials, the items evaluated were: confounding, participant selection, intervention classification, deviation from intended intervention, missing data, outcomes measurement, results report (Sterne *et al*., 2016).

### Statistical analysis

The measure of treatment effect was the pooled odds ratio of achieving a clinical pregnancy or live birth per ET for women in the Atosiban group, compared with women in the control group. For pooled data, we calculated summary test statistics using the Mantel-Haenzel method, using Rev-Man software, version 5.1. We based our meta-analyses on the number of women randomized, not on the number of women undergoing treatment.

We evaluated heterogeneity using the I^2^ test (Higgins *et al*., 2003) which indicates the proportion of variability across trials not explained by chance alone, and the *p*-value of X^2^ test of heterogeneity. Although interpreting the importance of inconsistency depends on other factors, the I^2^ values (e.g. *p*-value from X^2^ test, magnitude and direction of effects), the Cochrane Handbook suggests the following rough guide to interpreting the I^2^ values: low, moderate, and high to I^2^ values of 25%, 50%, and 75% test (Higgins *et al*., 2003).

A fixed effects model was used where no statistically significant heterogeneity was present, whereas in the presence of statistically significant heterogeneity, a random effects model was applied. Statistical significance was set at a *p* level of 0.05. The presence of publication bias was tested by using the Harbord-Egger’s test (Harbord *et al*., 2006).

### Subgroup analysis

If the overall I^2^ value for all trials was reduced when we separated the trials into subgroups according to source of bias, we used the subgroup results as primary. Otherwise, the pooled results from all trials would be used for our primary analysis, but with the results from the two subgroups also presented.

## RESULTS

### Search results

**Figure 1:**
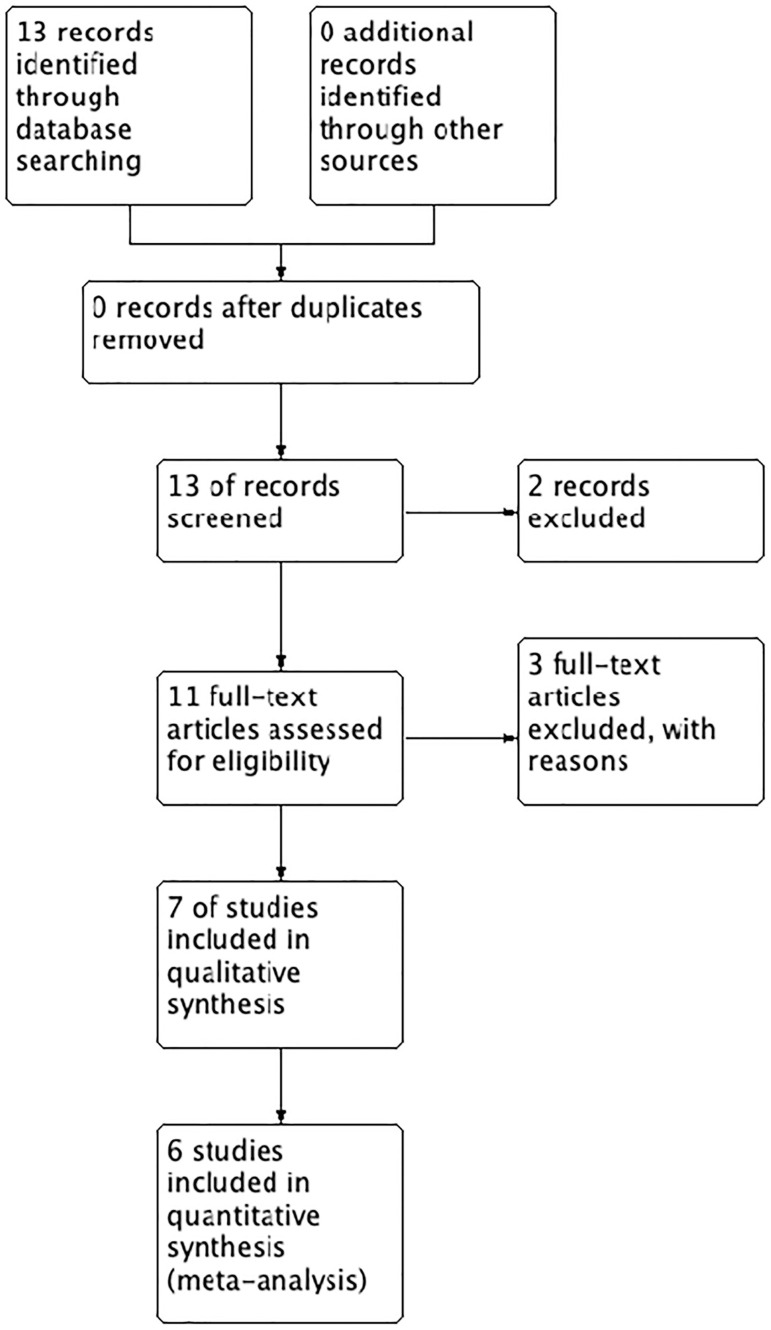
Flow diagram

The extensive literature search performed between the years 1990-2018 on Medline, EMBASE, yielded 13 publications. Of these, two were excluded based on the title and abstract. We then obtained the full text of the remaining 11 papers. See flow diagram in Figure 1.

### Included studies

Seven studies were considered in the synthesis, including 3 observational studies (Chou *et al*., 2011; He *et al*., 2016b; Lan *et al*., 2012) and 4 randomized controlled trials (He *et al*., 2016a; Hebisha *et al*., 2016; Moraloglu *et al*., 2010; Ng *et al*., 2014). The characteristics of the included trials are shown in Table 1.

**Table 1:** Characteristics of the studies included

Study, year	Design	Inclusion Criteria	Outcomes	Atosiban dose
Moraloglu *et al*., 2010	Prospective, randomized, placebo-controlled clinical study	Women undergoing intracytoplasmic sperm injection who had top-quality embryos	Clinical pregnancy rate per cycle and implantation rate	Intravenous atosiban 30 min before the embryo transfer with a bolus dose of 6.75 mg, and the infusion was continued with an infusion rate of 18 mg/h. After performing embryo transfer, the dose of atosiban was reduced to 6 mg/h and the infusion was continued for 2 h (total administered dose: 37.5 mg).
Chou *et al*., 2011	Retrospective cohort study	Repeated implantation failure (RIF)	Implantation rate, clinical pregnancy rate, live birth rate	Forty patients received a single bolus dose (6.75 mg, 0.9 mL/vial) of atosiban before ET (Group 2), and 30 patients received a bolus dose of 6.75 mg atosiban followed by infusion at 18 mg/hr. for 3 hours immediately after ET (Group 3).
Lan *et al*., 2012	Prospective cohort study	Women with repeated implantation failure	Uterine contraction, implantation rate (IR) and clinical pregnancy rate (CPR)	I.V. bolus of 6.75 mg at 30 min prior to embryo transfer followed by i.v. infusion at a rate of 18 mg/h for 1h and 6 mg/h for the subsequent 2h. The total dose administered was 36.75 mg.
Ng *et al*., 2014	Multi-center randomized double blind study	Consecutive subfertile women undergoing IVF treatment	The primary outcome measure was the live birth rate and the secondary outcome measures including positive pregnancy test, clinical pregnancy, ongoing pregnancy, miscarriage, multiple pregnancy and ectopic pregnancy rates.	I.V. Atosiban 30 min before the transfer with a bolus dose of 6.75 mg, and the infusion was continued at a rate of 18 mg/h for 1h. The dose of Atosiban was then reduced to 6 mg/h after embryo transfer and the infusion was continued for another 2h. Therefore, the total administered dose was 37.5 mg.
He *et al*., 2016a	Randomized, controlled clinical trial.	Women with endometriosis undergoing frozen–thawed embryo transfer	Implantation rate and pregnancy rate.	IV bolus of 6.75 mg at approximately 30 min before ET.
He *et al*., 2016b	Prospective cohort study	Patients undergoing IVF/ET using cryopreserved embryos	Uterine contraction, clinical pregnancy rate	I.V. bolus of 6.75 mg at about 30 min prior before ET.
Hebisha *et al*., 2016	Randomized controlled trial.	One hundred and eighty two women, prepared for intracytoplasmic sperm injection for male or tubal factor infertility, using long agonist protocol	Pregnancy rate, implantation rate.	7.5 mg Atosiban by slow IV injection

### Methods in the included studies

The study population included patients undergoing ICSI with the transfer of top-quality embryos (Moraloglu *et al*., 2010), regular IVF (Ng *et al*., 2014), women with repeated implantation failure (Chou *et al*., 2011; Lan *et al*., 2012), transfer of frozen/thawed embryos (He *et al*., 2016b), and transfer of frozen/thawed embryos in women with endometriosis (He *et al*., 2016a).

The intervention included the administration of a single bolus dose (Chou *et al*., 2011; Hebisha *et al*., 2016; He *et al*., 2016a;b) prior to the embryo transfer, or the administration of a bolus doses plus maintaining a continues dose (Lan *et al*., 2012; Moraloglu *et al*., 2010; Ng *et al*., 2014).

The outcomes evaluated included implantation rate (Chou *et al*., 2011; He *et al*., 2016a; Lan *et al*., 2012; Moraloglu *et al*., 2010), clinical pregnancy rate (He *et al*., 2016;b; Lan *et al*., 2012; Moraloglu *et al*., 2010; Chou *et al*., 2011; Hebisha *et al*., 2016) and delivery rate (Chou *et al*., 2011; Ng *et al*., 2014).

### Methodological quality of included studies

According to the guidelines suggested by the Cochrane Collaboration, the quality of most of the included studies was low to moderate due to unclear selection, performance and detection bias. Table 2 depicts the quality assessment of the included trials.

**Table 2:** Bias risks of the included RCT

Study, year	Domain 1	Domain 2	Domain 3	Domain 4	Domain 5	Domain 6	Domain 7
Moraloglu *et al*., 2010	High	High	Low	unknown	Low	Low	unknown
Ng *et al*., 2014	Unknown	Low	Low	unknown	Low	Low	Low
He *et al*., 2016a	Low	Low	unknown	unknown	Low	Low	Low
Hebisha *et al*., 2016	Unknown	unknown	unknown	unknown	Low	Low	unknown

1 Random sequence generation (selection bias)

2 allocation concealment (selection bias)

3 blinding of participants and personnel (performance bias)

4 blinding of outcome assessment (detection bias) (patient-reported outcome)

5 blinding of outcome assessment (detection bias) (all-cause main outcome)

6 incomplete outcome data (attrition data)

7 selective reporting (reporting bias)

### Result of the outcome measures

In total 1,025 women were allocated to Atosiban and 953 were allocated to a control group. Overall, we analysed four randomized controlled trials, including 1,292 patients, and two observational studies, including 686 patients.

**Figure 2:**
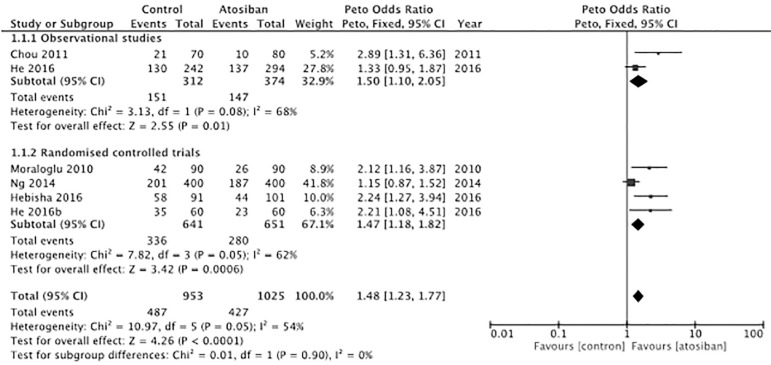
Forest plot

**Figure 3:**
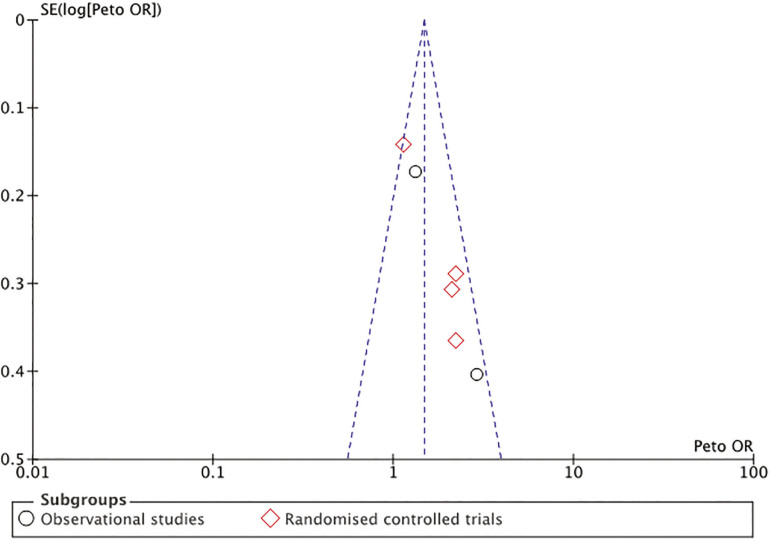
Funnel plot

In both, observational and randomized controlled studies, Atosiban was associated with an increased risk of clinical pregnancy. In the case of observational studies, the OR (95% CI) was 1.50 (1.10-2.05), with a moderate level of heterogeneity (I^2^ 68%, *p*=0.08). In the case of randomized controlled trials, the OR (95% CI) of clinical pregnancy was 1.47 (1.18-1.82), with moderate heterogeneity (I^2^= 62%, *p*=0.05). Figure 2 shows a forest plot with subgroup analyses for randomized and non-randomised controlled trials. To explore the heterogeneity, a funnel plot was drawn. The funnel plot (Figure 3) shows evidence of considerable symmetry.

## DISCUSSION

Atosiban was associated with an increase in the chance of clinical pregnancy. This increased probability was seen in both observational and randomized controlled studies, using different doses. ET is one of the crucial steps in ART, and the use of high-quality embryos together with the presence of an optimal intrauterine environment are the basic determinants of ET success (Dessolle *et al*., 2009). Fanchin *et al*. (1998a) demonstrated that uterine contractions occur during the course of ET. They reported that excessive uterine contractions can expel embryos from the uterus and that the frequency of uterine contractions was negatively correlated with implantation and clinical pregnancy rates. Since Atosiban is a combined oxytocin/vasopressin V1A antagonist, it works mainly by blocking oxytocin and vasopressin V1a receptors to decrease the frequency and amplitude of uterine contractions, which may enhance implantation and pregnancy rates (Pierzynski, 2011).

This is the most up-to-date review on this subject. The main strengths are the large sample of patients included, and an increased risk of pregnancy is supported by both, observational and randomized controlled studies. Furthermore, there was a positive effect of Atosiban regardless of the dose used. On the other hand, the main weakness of our study is that only six studies were found (since 2016, no new studies have been published) and the quality of the studies was relatively low.

In summary, we found that Atosiban was associated to an improvement in ART cycle outcomes, which might be of clinical significance, although, its administration requires a peripheral venous catheterization, longer hospitalization, and makes ET more expensive. Perhaps, the development of Nolasibam, an oral oxytocin receptor antagonist with the potential to decrease uterine contractions, will overrule these disadvantages in the near future.
